# A complete mitochondrial genome sequence of the wild two-humped camel (*Camelus bactrianus ferus*): an evolutionary history of camelidae

**DOI:** 10.1186/1471-2164-8-241

**Published:** 2007-07-18

**Authors:** Peng Cui, Rimutu Ji, Feng Ding, Dan Qi, Hongwei Gao, He Meng, Jun Yu, Songnian Hu, Heping Zhang

**Affiliations:** 1 Key Laboratory of Dairy Biotechnology and Engineering Ministry of Education, College of Food Science and Engineering, Inner Mongolia Agricultural University, Huhhot, China; 2 Key Laboratory of Genome Information and Sciences, Beijing Institute of Genomics, Chinese Academy of Sciences, Beijing, China; 3 Graduated School of the Chinese Academy of Sciences, Beijing, China; 4 School of Agriculture and biology, Shanghai Jiao Tong University, Shanghai, China

## Abstract

**Background:**

The family Camelidae that evolved in North America during the Eocene survived with two distinct tribes, Camelini and Lamini. To investigate the evolutionary relationship between them and to further understand the evolutionary history of this family, we determined the complete mitochondrial genome sequence of the wild two-humped camel (*Camelus bactrianus ferus*), the only wild survivor of the Old World camel.

**Results:**

The mitochondrial genome sequence (16,680 bp) from *C. bactrianus ferus *contains 13 protein-coding, two rRNA, and 22 tRNA genes as well as a typical control region; this basic structure is shared by all metazoan mitochondrial genomes. Its protein-coding region exhibits codon usage common to all mammals and possesses the three cryptic stop codons shared by all vertebrates. *C. bactrianus ferus *together with the rest of mammalian species do not share a triplet nucleotide insertion (GCC) that encodes a proline residue found only in the *nd1 *gene of the New World camelid *Lama pacos*. This lineage-specific insertion in the *L. pacos *mtDNA occurred after the split between the Old and New World camelids suggests that it may have functional implication since a proline insertion in a protein backbone usually alters protein conformation significantly, and *nd1 *gene has not been seen as polymorphic as the rest of ND family genes among camelids. Our phylogenetic study based on complete mitochondrial genomes excluding the control region suggested that the divergence of the two tribes may occur in the early Miocene; it is much earlier than what was deduced from the fossil record (11 million years). An evolutionary history reconstructed for the family Camelidae based on *cytb *sequences suggested that the split of bactrian camel and dromedary may have occurred in North America before the tribe Camelini migrated from North America to Asia.

**Conclusion:**

Molecular clock analysis of complete mitochondrial genomes from *C. bactrianus ferus *and *L. pacos *suggested that the two tribes diverged from their common ancestor about 25 million years ago, much earlier than what was predicted based on fossil records.

## Background

The family Camelidae has two Old World (tribe Camelina) species, bactrian camel (*Camelus bactrianus*) and dromedary (*C. dromedaries*), and four New World (tribe Lamini) species, guanaco (*Lama guanico*e), llama (*L. glama*), alpaca (*L. pacos*) and vicuna (*L. vicugna *or *Vicugna vicugna*) at present time [1,2]. The wild bactrian camel (*C. bactrianus ferus*) appears to be the only wild survivor of the Old World camel. According to the fossil record, Camelidae evolved in North America during the Eocene, approximately 40–45 million years ago [2], and the division between Camelini and Lamini occurred in North America about 11 million years ago [3,4]. In the late Tertiary (the epoch Pliocene) the species of Camelini and Lamini migrated from North America to South America and Asia separately, and their ancestors became extinct in North America subsequently. However, there have been very few molecular studies due to difficulties in either obtaining enough DNA samples or acquiring enough sequence information. Previous molecular studies, mainly focusing on the sequence of mitochondrial *cytochrome b *gene, have made significant contributions to understanding the evolutionary history of Camelidae [2], and yet there has not been any significant comparative studies on the evolutionary relationship between Camelini and Lamini.

Mitochondrial DNA (mtDNA) has been proven useful for studying evolutionary relationships among animal species, due to its conservativeness in protein-coding sequences, high substitution rate in its non-coding sequences, and lack of genetic recombination [5,6]. To investigate the evolutionary relationship between Camelini and Lamini, we have made an unprecedented effort to obtain adequate samples from the wild two-humped camel, sequenced its mitochondrial genome completely, and carried out detailed sequence and evolutionary analyses.

## Results

### Genome organization

Since mammalian mitochondrial genome sequences are very similar, we designed a set of PCR primers based on highly conserved sequences of an alignment with full-length mitochondrial genomes from the available public data, including those of cow, deer, sheep, pig, and lama (Table 1). We sequenced some of the PCR-amplified DNA segments first to obtain as much authentic sequences as possible from the wild two-humped camel, and subsequently designed new primers according to the newly acquired sequences. We collected 119 raw sequence traces with an average length of 521 bp at a quality value of Q20, which cover the entire genome four folds.

**Table 1:** PCR primers used for the experiment

Primer	Sequence(5' -3')	Strand
12s-121^a^	GGAGCTGGTATCAAGCAC	upper
12s-942	CAGTATGCTTACCTTGTTAC	lower
16s-136	GCTATAGAGAAAGTACCGTAAG	upper
16s-648	CTCATATTAACATTATTGCTTC	lower
16s-1053	ACTGTCTCTTACTTCCAATC	upper
16s-1478	ATAGATAGAAACCGACCTG	lower
nd1-85	AGTAGAACGAAAAGTCCTAG	upper
nd2-791	TTAATTCTTGGATGATTATTC	lower
cox2-380	ACTCCTATATAATCCCAACATCAG	upper
atp6-449	GCTAGGGCTACTGGTTGAATAA	lower
atp6-301	ACACCAACTACACAACTATCAATAA	upper
cox3-188	CTTGGAAGGTGCTTTCTC	lower
cox3-205	GAGAAAGCACCTTCCAAG	upper
cox3-717	TCTACGAAATGTCAATATCAG	lower
nd4-379	TCCTATTTGAAGCAACACTAG	upper
nd4-804	ATTGAGCTGGTTATAATTATG	lower
nd5-767	CTCAAGCACAATAGTAGTAGCAGG	upper
cytb-830	ATTGATCGTAAGATTGCGTATG	lower
cytb-419	CCATGAGGACAAATATCATT	upper
cytb-1001	CCTCCAATTCATGTGAGTG	lower

^a^The numbers refer to the position of PCR primers (3') in mitochondrial genes.

**Figure 1:**
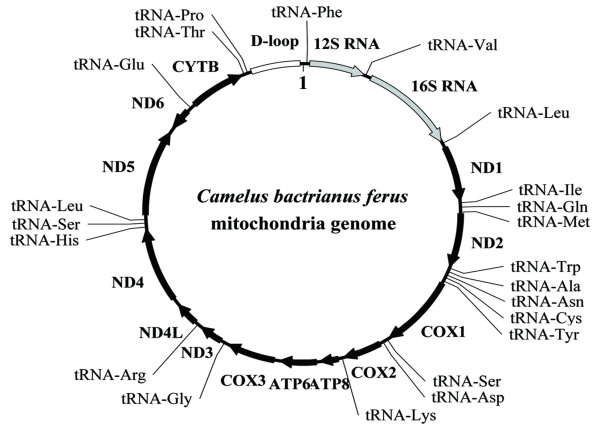
The mitochondrial genome of *C. bactrianus ferus *(16,680 bp). Protein-coding genes (black) and rRNA genes (light-gray) are identified by arrows. The gene *nd6 *is transcribed in the opposite direction as relative to the rest in the cluster. tRNA genes are depicted with their corresponding amino acids.

The full-length mitochondrial genome is 16,680 bp in length [GenBank: EF212038], which is 28 bp longer than that of *L. pacos*. The minor length variation mainly occurred in the tandem repeat (ACGTAC)_n _of the control region. The gene order and content are identical to those of other mammals (Figure 1); it harbors 13 protein-coding genes (three subunits of the cytochrome c oxidase, seven subunits of the NADH ubiquinone oxidoreductase complex, one subunit of the ubiquinol cytochrome b oxidoreductase complex, and two subunits of ATP synthases), the small and large ribosomal RNA genes, and 22 tRNA genes (Table 2). The replication origin of the light strand within a tRNA gene cluster was also unambiguously identified.

**Table 2:** Organization of *Camelus bactrianus ferus *mitochondrial genome

Gene/element	Strand	Position	Start	Stop	Size	GC %
tRNA-Phe	H	1–67	-	-	67	41.79
12S rRNA	H	68–1034	-	-	967	43.54
tRNA-Val	H	1035–1100	-	-	66	38.57
16S rRNA	H	1101–2666	-	-	1566	38.25
tRNA-Leu	H	2667–2741	-	-	75	42.67
*nd1*	H	2745–3701	ATG	TAA	957	45.24
tRNA-Ile	H	3701–3769	-	-	69	30.43
tRNA-Gln	L	3767–3839	-	-	73	35.71
tRNA-Met	H	3841–3909	-	-	69	45.25
*nd2*	H	3910–4953	ATA	TAG	1044	37.07
tRNA-Trp	H	4966–5019	-	-	54	43.24
tRNA-Ala	L	5025–5093	-	-	69	43.48
tRNA-Asn	L	5095–5167	-	-	73	39.21
OL	-	5157–5211	-	-	55	52.75
tRNA-Cys	L	5201–5267	-	-	67	40.58
tRNA-Tyr	L	5268–5336	-	-	69	41.30
*cox1*	H	5338–6885	ATG	TAG	1548	44.12
tRNA-Ser	L	6887–6957	-	-	71	39.44
tRNA-Asp	H	6962–7028	-	-	67	40.68
*cox2*	H	7029–7712	ATG	TAA	684	40.94
tRNA-Lys	H	7716–7782	-	-	67	47.76
*atp8*	H	7784–7987	ATG	TAA	204	34.80
*atp6*	H	7945–8625	ATG	TAA	681	39.20
*cox3*	H	8625–9403	ATG	T . .	779	47.75
tRNA-Gly	H	9409–9478	-	-	70	30.43
*nd3*	H	9476–9825	ATA	TA .	350	43.14
tRNA-Arg	H	9826–9893	-	-	68	44.93
*nd4l*	H	9894–10190	ATG	TAA	297	40.74
*nd4*	H	10184–11570	ATG	T . .	1387	42.39
tRNA-His	H	11562–11630	-	-	69	34.78
tRNA-Ser	H	11631–11689	-	-	59	44.12
tRNA-Leu	H	11691–11760	-	-	70	39.73
*nd5*	H	11761–13581	ATA	TAA	1821	41.30
*nd6*	L	13565–14092	ATG	TAA	528	41.48
tRNA-Glu	L	14094–14162	-	-	69	34.78
*cytb*	H	14167–15306	ATG	AGA	1140	43.25
tRNA-Thr	H	15307–15375	-	-	69	34.80
tRNA-Pro	H	15375–15440	-	-	66	43.84
Control region	H	15441–16687	-	-	1247	46.75

H and L refer to heavy and light strains of the mitochondrial genome, respectively.

### Protein-coding genes

We carefully annotated 13 protein-coding genes (Table 2), including start and stop codons (ten ATGs, three ATAs, seven TAAs, two TAGs, and one AGA). Three of them, *cox3*,*nd3*, and *nd4*, do not have normal stop codons but have the cryptic components, T, TA, and T, respectively. The possession of such noncanonical start and stop codons is shared by most of the vertebrate mitochondrial genomes. These T or TA stop codon components are supplemented via posttranscriptional polyadenylation later in the cellular translation process [7]. Another common feature among vertebrate mitochondrial genomes is the existence of overlapping genes; for instance, *ATP6 *and *ATP8 *share a 42-bp sequence.

The mitochondrial genome of the wild camel shares its genetic code with other vertebrates (Table 3). In this mtDNA, the codon CTA for leucine is the most frequently used (277 times) and the codon CGT for arginine is the least frequently found (7 times) even though both amino acids are separately encoded by six and four codons in the canonical genetic code. The codons ending with A or T are used more frequently than those ending with G or C since it has a high A+T (57.9%) content similar to what have been found among other mammals.

**Table 3:** Comparison of codon usage among *Camelus bactrianus ferus *and selected vertebrates

AA^a^	Codon	Camel	Alpaca	Dog	AA	Codon	Camel	Alpaca	Dog
G	GGC	67	63	51	M	ATA	177	195	201
	GGA	92	91	93		ATG	75	58	47
	GGG	36	30	28	D	GAT	28	31	28
A	GCT	57	52	63		GAC	41	42	41
	GCC	109	103	86	E	GAA	61	66	73
	GCA	88	88	89		GAG	35	28	21
	GCG	12	9	14	N	AAT	59	73	66
V	GTT	46	43	49		AAC	92	71	91
	GTC	49	60	27	Q	CAA	67	78	68
	GTA	80	84	65		CAG	23	15	21
	GTG	33	15	24	K	AAA	77	83	81
L	TTA	97	97	117		AAG	19	18	19
	TTG	32	15	28	R	CGT	7^c^	6	6
	CTT	72	68	96		CGC	14	16	10
	CTC	97	93	90		CGA	39	39	45
	CTA	227^b^	279	230		CGG	8	4	4
	CTG	68	46	33	H	CAT	28	28	38
I	ATT	179	167	174		CAC	63	65	57
	ATC	134	151	168	P	CCT	54	43	64
S	TCT	55	54	68		CCC	81	84	62
	TCC	75	69	74		CCA	49	61	60
	TCA	71	80	78		CCG	12	10	7
	TCG	10	8	12	F	TTT	103	122	108
T	ACT	60	70	75		TTC	133	108	127
	ACC	97	89	90	Y	TAT	69	68	69
	ACA	126	142	120		TAC	68	73	74
	ACG	34	19	18	W	TGA	88	90	94
C	TGT	11	9	4		TGG	17	15	10

^a ^The one-letter code for amino acids (AA) was used;

^b ^the most frequently used codon;

^c ^the least frequently used codon.

### Transfer RNA genes

**Figure 2:**
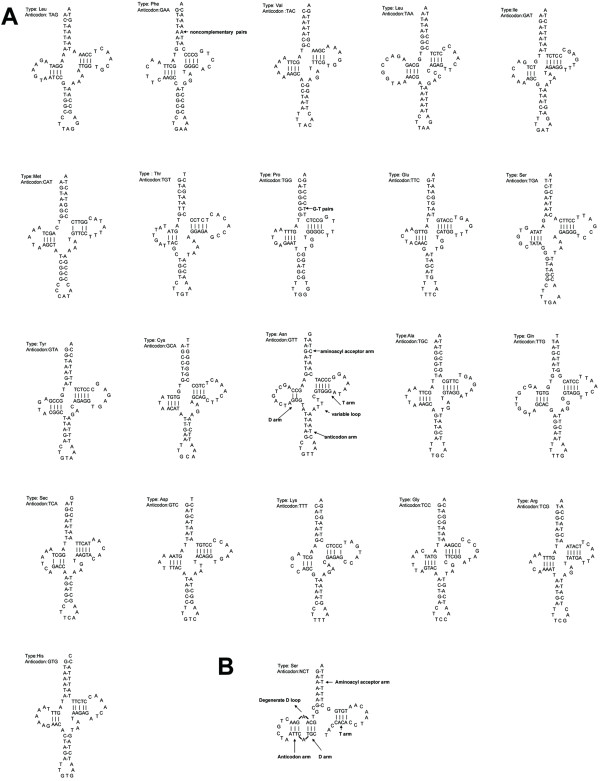
Predicted secondary structures of 22 mitochondrial tRNA sequences from the wild camel. All tRNAs were folded into secondary structures based on the "least free energy" principle. (A) 21 tRNAs exhibit cloverleaf structures and possess anticodons. The secondary structure of tRNA^Asn ^was shown (bold) as an example for the cloverleaf structure that usually has 7 bp in the aminoacyl stems, 4 bp or 5 bp in the TψC and anticodon stems, and 4 bp or 3 bp in the DHU stems. tRNA stem regions include some non-complementary and T:G base pairings. (B) tRNA^ser ^can not form the appropriate dihydrouridine loop and does not possess the anticodon for AGN.

We identified 22 tRNA genes in mtDNA of the wild camel; 21 of them were predicted capable of folding into the cloverleaf structure and possess anticodons that match the vertebrate mitochondrial genetic code (Figure 2A). Only one of them, tRNA^ser ^lacks the appropriate sequence elements for forming an orthodox cloverleaf structure as well as both dihydrouridine loop and the anticodon (Figure 2B). Although it has been predicted that this tRNA^ser ^gene (for AGN) may be a pseudogene as observed among other metazoan mitochondrial genomes and some of them were proven inactive [8], efforts have been made to uncover underlying molecular details as to how serine residues are actually added into peptide chains with this "bizarre" tRNA [9].

### Non-coding sequences

**Figure 3:**
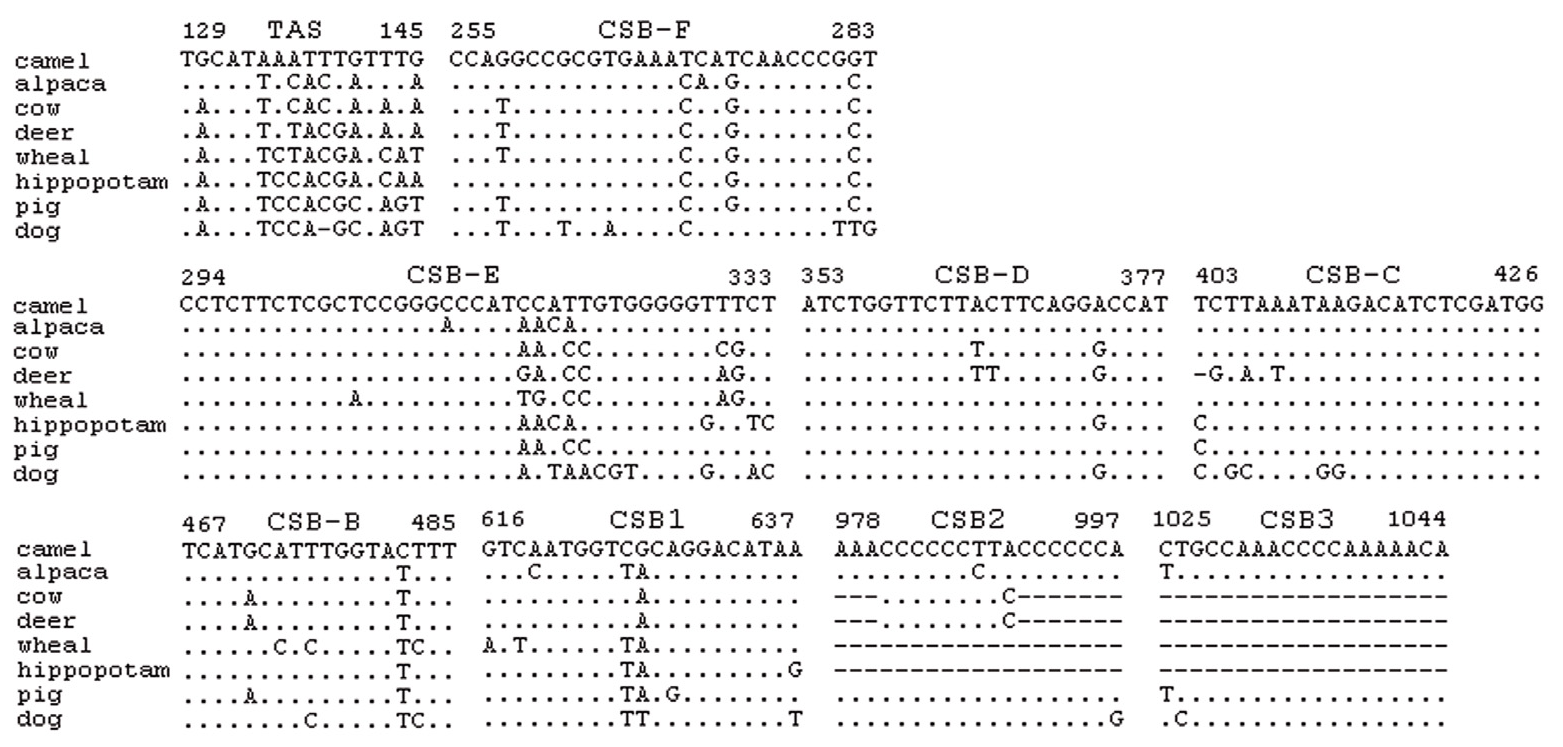
Alignment of termination-associated sequences (TAS) and eight putative conserved sequence blocks of CBS (1–3) and CBS (B-F). The bactrian camel sequence was used as the consensus sequence, orientated from 5' to 3' on the heavy strand. Dots, dashes, and numbers indicate nucleotide identity, indels, and positions of the first and the last nucleotide for each identified region, respectively.

Among vertebrate mitochondrial genomes, most of the non-coding sequences are part of the control region, about 700–1,300 bp in length, responsible for the regulation of replication and transcription [10]. The control region shows extensive variability across taxonomic groups and even among related species, but its sequence elements related to regulatory functions are known to be highly conserved. We annotated the 1,247-bp control region and regulatory domains; at the 5' end, we found termination-associated sequence (TAS) motifs that act as a signal for terminating synthesis of the D-loop strand [11,12]. The sequence between bactrian camel and alpaca showed less similarity as expected (Figure 3). We also discovered all eight putative conserved sequence blocks (CSB1-3 and B-F) that are important to the regulation of mtDNA replication [12]; among them, CSB1 and CSBC-F are conserved among mammals, whereas CSB2 and CSB3 are absent from some of them.

### Mitochondrial DNA variations in camelidae

To investigate mitochondrial DNA variations in Camelidae, we compared the two complete sequences from the wild camel and alpaca; the comparison yielded 1,522 transitions and 389 transversions when excluding the highly variable control region. We also identified 26 single nucleotide indels (insertions or deletions) from non-coding RNA sequences. The most striking observation was a codon indel (GCC) encodes proline residue found in the *nd1 *gene between *C. bactrianus ferus *and *L. pacos*. This indel, upon further scrutiny, is actually an insertion in the *L. pacos *mtDNA but absolutely absent in the rest of mammalian mtDNA collection. In other words, it is lineage-specific to the New World *L. pacos*, suggesting that an insertion event occurred after the Old and New World Camelidae split. Furthermore, our comparative analyses showed a relatively lower ratio of nonsynonymous substitution (K_a_) to synonymous substitution (K_s_) in *cox1*, *cox2*, *cox3*, and *cytb *genes (Table 4), whereas a large number of nonsynonymous substitutions were observed in *nd2*, *nd3, nd4, nd4L*, and *atp6 *gene sequences, especially in the *atp8 *gene. This result indicates that the ND family and ATP synthase genes may evolve faster than other genes among the 13 protein-coding ones. This pattern is not universal among all mammalian mitochondrial genomes and suggests its functional relevance for mitochondrial biology. To our surprise, the *nd1 *gene that harbors the codon insertion in *L. pacos *does not appear to have higher mutation rate as compared to other ND family genes in its mtDNA.

**Table 4:** The ratio of nonsynonymous substitution (K_a_) vs. synonymous substitution (K_s_) among the mitochondrial genes

Gene	Ka	Ks	Ka/Ks
*nd6*	0.056	1.248	0.045
*atp6*	0.040	0.598	0.067
*atp8*	0.162	0.557	0.290
*cox1*	0.009	0.866	0.010
*cox2*	0.024	1.123	0.021
*cox3*	0.026	1.601	0.016
*cytb*	0.051	1.271	0.040
*nd1*	0.023	1.847	0.012
*nd2*	0.043	0.665	0.064
*nd3*	0.063	0.929	0.068
*nd4*	0.028	1.008	0.028
*nd4l*	0.040	0.874	0.046
*nd5*	0.056	1.123	0.050

### Phylogenetic analyses

**Figure 4:**
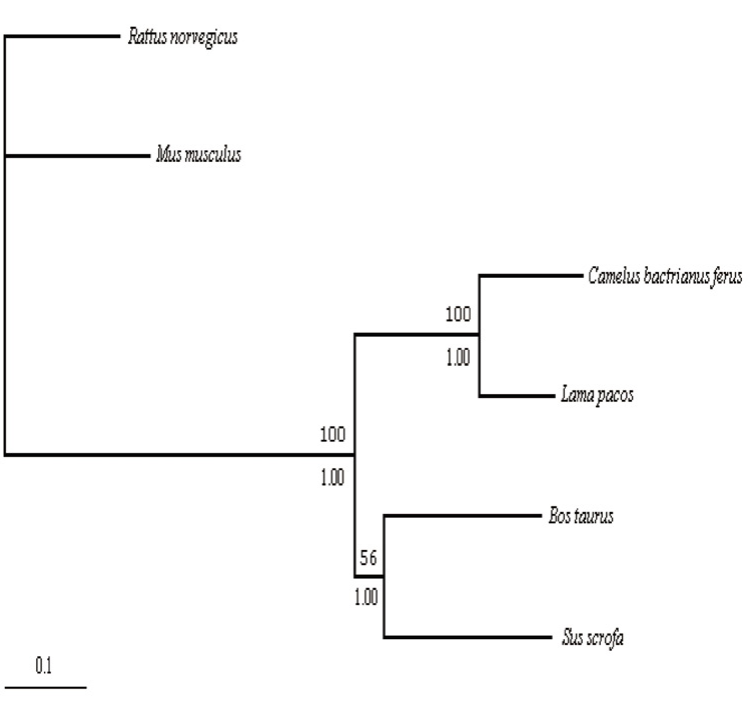
The phylogenic relationship between bactrian camel and alpaca based on maximum likelihood analysis (without the control regions). To estimate the divergence time, cow and pig were taken as an ingroup calibration point, while the mouse and rat were taken as an outgroup calibration point. All nodes were supported by the bootstrap value (1000 replications) and posterior probability on the nodes (above the branch is the bootstrap value, and below the branch is posterior probability). The scale bar indicates 0.1 substitutions.

To date the divergence time between Camelini and Lamini, we aligned complete mtDNA sequences of the wild camel and alpaca as well as other selected mammals. We then constructed a phylogenetic tree from these aligned sequences by using those of rat and mouse as outgroup (Figure 4). Since the control region of mtDNA has a high incidence of homoplasy [5,13], we excluded it for this analysis. Similar topology was obtained with both neighbor-joining and maximum likelihood methods. Bootstrap values and Bayesian posterior probabilities were showed for all clades.

Estimating divergence time can be done with or without assuming a constant rate evolutionary rate among all compared clades. Since evolutionary rate of Camelidae mitochondrial genomes has not been well-studied, we tested the rate constancy in Camelidae using mtDNA excluding the control region. We first performed Tajima's relative rate test, using cow as an outgroup. Molecular clock analyses are highly sensitive to evolutionary distance between main clades and the choice of an outgroup is of importance [14]. The result rejected the assumption of a constant rate of change among the camelid mitochondrial genomes (p < 0.01, unpublished data). To further confirm this conclusion, we used likelihood ratio method for a similar test (Materials and Methods), and showed obvious differences in the evolutionary rate between the two tribes (without rate constancy LnL1 = -38557.259972; with rate constancy LnL2 = -38550.07554; P < 0.01).

Since these studies pointed out that the assumption of rate constancy might not be appropriate for estimating divergence time between Camelini and Lamini, we used a heuristic rate smoothing procedure for ML-based estimates [15], which takes into account evolutionary rates among different branches of the tree. We used two calibration points, 65 and 14 million years, which were the estimated timing for the divergence of cow-pig (artiodactyl calibration) and mouse-rat (outgroup calibration), respectively [16,17]. Average rates for the wild camel and alpaca were 1.2% per million years and 0.9% per million years, respectively. Therefore, the divergence time between Camelini and Lamini was estimated to be 25 million years, significantly earlier than what was estimated based on fossil records (11 million years). This result suggested that the divergence of the two tribes might occur in the early Miocene [2,4].

**Figure 5:**
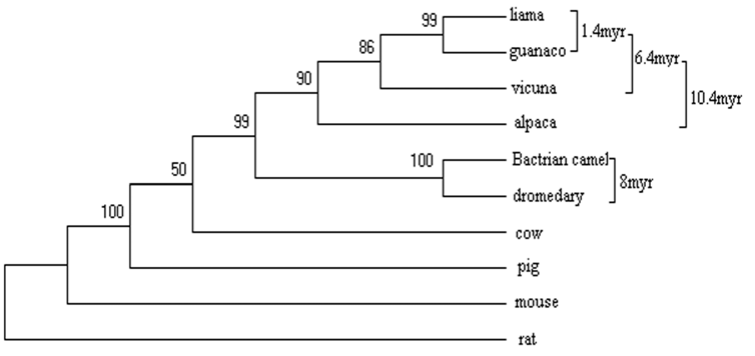
Phylogeny of the Camelidae species based on maximum likelihood analysis of *cytb *sequences. To estimate the divergence time, pairwise comparisons of cow-pig and mouse-rat were taken as the ingroup and outgroup calibration points, respectively. All nodes were supported by the bootstrap value (1000 replications) on the nodes.

We also constructed a phylogenetic tree based on the *cytb *gene sequences from the wild camel and other camelids, and looked into other possibilities (Figure 5). Similarly, we used the two calibration points, 65 and 14 million years, and performed a maximum likelihood procedure to estimate divergence time. In tribe Camelini, the divergence time of bactrian camel and dromedary was estimated as 8 million years, whereas in tribe Lamini, alpaca diverged from the other three species about 10 million years ago. Guanaco and llama diverged from their common ancestor in the early Pleistocene, 1.4 million years ago.

## Discussion

Although the mitochondrial genome of the wild two-humped camel is highly similar to those of other mammals, there are substantial differences in the mtDNA evolutionary rate for different taxonomic groups. Relative rate tests demonstrated the evolutionary rate also varies between bactrian camel and other mammals (Table 5). The difference within a taxonomic group is as significant as among groups. For example, the rate for bactrian camel is significantly different from that for alpaca, so does the rate between bactrian camel and cow.

**Table 5:** Relative rate test for pairs among vertebrate lineages

Taxon pairs	Outgroup	x^2^	p
camel	cow	16.87	< 0.001
alpaca			
camel	pig	40.38	< 0.001
cow			
camel	cow	46.03	< 0.001
pig			
camel	pig	25.11	< 0.001
deer			
camel	pig	30.19	< 0.001
whale			

The divergence time for Camelini and Lamini was estimated to be 25 million years or in the early Miocene. This estimate is significantly earlier than what were suggested based on the fossil record in North America [2,4]. In tribe Camelini, we estimated that speciation of bactrian camel and dromedary may have begun 8 million years ago. If the time of the camel migrating from North America to Asia estimated based on the fossil material is correct (3 million years) [2,4], the speciation of the genus Camelus may have occurred in North America. In tribe Lamini, alpaca is an older lineage that evolved to become the current species about 10 million years ago. In contrast, guanaco and llama diverged from their common ancestor much later, about 1.4 million years ago in the early Pleistocene; this divergence occurred after Lamini species migrated from North America to South America [2,4].

## Conclusion

The mtDNA of *C. bactrianus ferus *is very similar to that of other mammals. It contains 13 protein-coding, two rRNA, and 22 tRNA genes as well as a typical control region. Molecular clock analyses on complete mitochondrial genomes from *C. bactrianus ferus *and *Lama pacos *suggested that the two tribes diverged from their common ancestor about 25 million years ago, much earlier than what were suggested based on fossil record. The evolutionary history of Camelidae, reconstructed by using *cytb *sequences, suggested that the speciation of bactrian camel and dromedary may have occurred in North America before they migrated to South America and subsequently left the continent.

## Methods

### Specimens, DNA amplification, and sequencing

The samples (ear punches) from the wild two-humped camel were collected in the Gobi area (the Trans-Altai Gobi of Mongolia as described by Sanduin 2002) of Mongolia in 2006. Genomic DNA was extracted according to the proteinase K/phenol extraction method. A PCR-Based approach for mitochondrial genome sequencing was used ([18]. The PCR primers used for initial amplification were designed based on conserved known mammalian sequences from the public databases, and new primers were also designed according to the newly generated sequence data for finishing the entire genome.

The standard PCR was conducted in a 25 μl reaction volume that contains 1–2 U Taq DNA polymerase, 10 mM Tris-HCl (pH 8.3), 0.25 mM dNTPs, 0.2–2 mM BSA, 1.5–2.5 mM MgCl_2_, 20 pM of each primer, and about 10 ng camel genome DNA. The PCR reaction conditions were set as: 94°C for the first 5 min, followed by 35 cycles of 94°C denaturation for 30s, 50°C annealing for 30s, and 72°C extension for 45s. The PCR were accompanied by negative controls containing the reaction solutions without DNA.

The thermo-cycling sequencing reaction was performed in a final volume of 24 μl containing 16 μl DYEnamic ET Terminator Sequencing Kit premix, 10 pM sequencing primers, and 500 μg DNA. The reaction conditions were 95°C for 2 min, followed by 35 cycles of 95°C denaturation for 15s, 50°C annealing for 15s, and 60°C extension for 90s. The amplified DNA fragments were sequenced on ABI-3730 DNA sequencer. The primers for PCR reaction were used for sequencing. DNA sequences were assembled by using the software package phred/phrap/consed/[19,20] on a PC/UNIX platform. The mitochondrial sequences were annotated with BLAST tools, and tRNA genes and their secondary structures were identified according to tRNAscan-SE [21].

### Phylogenetic analysis

The complete mitochondrial genome of *L. pacos *[GenBank:Y19184],*Bos taurus *[GenBank:AY526085], *Sus scrofa *[GenBank:AF034253], *Mus musculus *[GenBank:AY172335], *Rattus norvegicus *[GenBank:AY172581] and *cytb *gene sequences [GenBank:EF212038, GenBank:EF212037, GenBank:AY535258, GenBank:U06430, GenBank:U06429, GenBank:Y19184, GenBank:AY526085, GenBank:AF034253, GenBank:AY172335, GenBank:AY172581] used for phylogenetic analysis and other mammals used for comparison and PCR primer designs were obtained from the NCBI database. Multiple alignments were performed with the CLUSTAL W [22]. Alignments of all protein-coding genes were used to estimate variation rates at synonymous (d_S_) and nonsynonymous (d_N_) sites with a maximum likelihood (ML) method [15] implemented in Ka_Ks_Calculator [23].

The phylogenic trees were reconstructed separately according to the neighbor-joining method implemented in MEGA [24] and the maximum likelihood method implemented in PHYLIP [25]. The reliability of the branches was assessed by bootstrap analysis (1000 replications). Bayesian posterior probability of phylogeny was done with MrBayes (mcmc ngen = 1000000) [26]. Two different prior models were used: the General Time Reversible model and the HKY model.

Rate constancy was tested with the Tajima's [27] and likelihood ratio tests. The likelihood ratio test was done with the baseml program in PAML. Two parameters of no molecular (clock = 0 in baseml) and the local clock (clock = 2 in baseml) were used to test molecular clock. When bactrian camel and alpaca were tested, cow was used as outgroup. To estimate the divergence time, a heuristic rate smoothing procedure for ML estimates was used as implemented in PAML [15]

## Abbreviations

*cox1*, *cox2*, *cox3 *– cytochrome oxidase subunit I, II, and III protein genes; *atp6, atp8 *– ATP synthase subunit 6 and 8 genes; *nad1*, *nad2*, *nad3*, *nad4*, *nad5, nad6, nad4L *– NADH dehydrogenase subunit 1–6, 4L genes; *cytb *– cytochrome b gene; mtDNA- mitochondrial DNA; AA – amino acids.

## Authors' contributions

HPZ and SNH were primarily responsible for the design and conducting this study. PC and FD were responsible for determining and assembling mtDNA sequences. RMTJ and JNG performed data analysis. PC and JY wrote the manuscript. HM and HWG were responsible for sample collection. All authors have read and approved the final manuscript.
